# Comparison of the Effects of Intermittent Energy Restriction and Continuous Energy Restriction among Adults with Overweight or Obesity: An Overview of Systematic Reviews and Meta-Analyses

**DOI:** 10.3390/nu14112315

**Published:** 2022-05-31

**Authors:** Jun Wang, Fang Wang, Hongxiu Chen, Li Liu, Shuai Zhang, Wenjing Luo, Guan Wang, Xiuying Hu

**Affiliations:** West China School of Nursing, Sichuan University/Innovation Center of Nursing Research, Nursing Key Laboratory of Sichuan Province, National Clinical Research Center for Geriatrics, West China Hospital, Sichuan University, No.37 Guoxue Lane, Wuhou District, Chengdu 610041, China; 15250062923@163.com (J.W.); bio_wangf@163.com (F.W.); chenhongxiu@stu.scu.edu.cn (H.C.); liuli837@wchscu.cn (L.L.); sshuai0326@163.com (S.Z.); luowenjing23@163.com (W.L.)

**Keywords:** overview, intermittent energy restriction, continuous energy restriction, obesity

## Abstract

There is considerable heterogeneity across the evidence regarding the effects of intermittent energy restriction and continuous energy restriction among adults with overweight or obesity which presents difficulties for healthcare decision-makers and individuals. This overview of systematic reviews aimed to evaluate and synthesize the existing evidence regarding the comparison of the two interventions. We conducted a search strategy in eight databases from the databases’ inception to December 2021. The quality of 12 systematic reviews was assessed with A Measurement Tool to Assess Systematic Reviews 2 (AMSTAR 2) and the Grading of Recommendations Assessment, Development and Evaluation (GRADE). One review was rated as high quality, 1 as moderate, 4 as low, and 6 as critically low. A meta-analysis of the original studies was conducted for comparison of primary intermittent energy restriction protocols with continuous energy restriction. Intermittent energy restriction did not seem to be more effective in weight loss compared with continuous energy restriction. The advantages of intermittent energy restriction in reducing BMI and waist circumference and improvement of body composition were not determined due to insufficient evidence. The evidence quality of systematic reviews and original trials remains to be improved in future studies.

## 1. Introduction

According to the definition of obesity given by the World Health Organization (WHO), body mass index (BMI) ≥30 kg/m^2^ is obesity, and 25 < BMI < 30 kg/m^2^ is overweight in adults [1]. Studies from different countries show that the number of adults with overweight or obese has been rising rapidly [2,3,4,5]. It is estimated that 38% and 20% of adults will be overweight and obese by 2030, respectively [6]. Overweight and obesity result in a great burden for both individuals and society: To be specific, it contributes to the increased risk of many diseases including cardiovascular disease (CVD), hypertension, hyperlipidemia, diabetes, and even certain cancers, which impose huge social, medical, and economic burdens [7,8,9,10]. The Global Burden of Disease study reported 4.7 million people died prematurely in 2017 due to obesity [11]. Hence, efforts to provide more effective and economical strategies for weight loss in individuals with overweight or obesity are required to reduce the burden of obesity-related diseases.

Calorie restriction (CR) is a primary popular non-pharmaceutical intervention for weight loss in individuals with overweight or obesity [12,13,14,15], which includes two main forms: intermittent energy restriction (IER) and continuous energy restriction (CER). IER is characterized by periods of marked energy restriction combined with a normal energy intake [16]. The primary popular IER protocols would be 5:2 diet, alternate-day fasting (ADF), and time-restricted feeding (TRF) [17]. CER is a diet strategy of reducing 15–40% of the baseline calorie needs for a continuous period for weight loss [18]. Since the appearance of IER after CER, there has been increasing controversy about the effects of IER and CER among adults with overweight or obesity on weight loss, waist circumference, and body composition [19,20,21].

In recent years, many systematic reviews and meta-analyses have focused on whether IER could replace CER on the benefits and advantages of reducing body weight, waist circumference, and improvement of body composition. Although CR has been identified as an effective life intervention [22], there is a lack of relevant evidence addressing the issue: which type of CR intervention has more advantages in weight loss, reducing waist circumference, and improvement of body composition? There is abundant evidence targeting the comparison of the effects of IER and CER on those indicators; however, the quality of evidence is discrepant and the conclusions remain inconsistent and unclear [19,23,24,25,26].

The evidence is not sufficient to draw conclusions and a more comprehensive evaluation is necessary, and as such, we implemented an overview of systematic reviews to integrate and sort out the consistent and different parts for improving the integrity and accuracy of the evidence. It will provide evidence on choosing more suitable and beneficial diet strategies for adults with overweight or obesity and offer evidence to support the actions of reducing the burden of obesity and its related diseases.

## 2. Materials and Methods

### 2.1. Protocol and Registration

This overview of systematic reviews was conducted with the Statement of Preferred Reporting Items for Systematic Review and Meta-analysis (PRISMA) [27] (Supplementary Materials S1) and was registered at the International Prospective Register of Systematic Reviews (PROSPERO) with the number CRD42021272442.

### 2.2. Deviations from the Registered Protocol

There are some deviations from the registered protocol that needs to mention. First, we decided not to report cardiovascular diseases related indicators since the unclear statements in evidence with low quality and heterogeneity of the studies. Second, we added time-restricted feeding (TRF) as a primary protocol of IER, and periodic fasting was deleted because its main forms were the 5:2 diet and alternate-day fasting (ADF). Third, the restriction of intervention duration was canceled for more comprehensive evidence. Fourth, we decided to conduct an additional meta-analysis rather than narrative text to provide more convincing evidence.

### 2.3. Inclusion and Exclusion Criteria

#### 2.3.1. Type of Included Reviews

We included systematic reviews and meta-analyses of randomized controlled trials that compared IER and CER in adults with overweight or obesity. The non-systematic narrative reviews, individual studies, case reports, case series, editorials, and clinical guide publications were out of consideration. All included reviews in this work met the following items:

- A systematic search strategy was used to guide literature retrieval;
- The criteria for included studies were explicit;
- More than two databases were searched;
- The outcomes of data extraction and quality assessment of included studies were finished and presented.

#### 2.3.2. Type of Intervention

The 5:2 diet, ADF, and TRF were included in our study as primary IER protocols. Definitions are as follows:

- 5:2 diet [28]: It consists of 2 days (consecutive or non-consecutive) of complete fasting or lower calorie intake than needed plus ad libitum eating on the other days per week;
- Alternate-day fasting, ADF [17]: It involves alternating ad libitum feeding days with fasting days. On fasting days, one is allowed to have a lower calorie intake than needed or complete fasting;
- Time-restricted feeding, TRF [17]: It involves following the same eating routine each day, with a certain number of hours designated as the fasting window and the remaining hours as the feeding window.

The comparison is with CER [18] or daily calorie restriction, which reduces 15–60% of the baseline calorie needs each day.

#### 2.3.3. Participants

We included adults with overweight or obesity (BMI ≥ 25 kg/m^2^, age ≥ 18 years old). The following targeting populations were excluded: participants with neuropsychiatric disease; participants undergoing or previously received bariatric surgery; participants who are pregnant or breastfeeding; and participants taking medication associated with weight loss or weight gain.

#### 2.3.4. Outcomes

The outcomes of this study are as follows:

- Bodyweight (kg);
- Body mass index (kg/m^2^);
- Waist circumference (cm);
- Fat mass (kg);
- Fat-free mass (kg).

### 2.4. Search Methods

Eight electronic databases were searched from databases inception to December 2021: The Cochrane Library of Systematic Reviews, Clinical Trials, PubMed, Medline (Ovid), Embase (Ovid), Scopus, PROSPERO, and Web of Science. The search strategy was presented in Supplementary Materials S2. The search phrases consist of medical subject heading (MeSH) terms and free-text words related to “intermittent energy restriction”, “5:2 diet”, “alternate day fasting”, “time-restricted feeding”, “continuous energy restriction”, “overweight”, “obesity”, “systematic review”, and “meta-analysis”.

### 2.5. Selection and Data Extraction

After deleting duplicates, two authors independently screened the remaining records according to the title and abstract and then selected the potentially qualified systematic reviews or meta-analyses. Then the two authors evaluated the potentially qualified literature in full text independently for including qualified records in this work. The following data in reviews were extracted: author, published year, the number of included studies, population type, sample size, age, BMI, intervention, comparison, and outcomes. *p*-values were also extracted, and the differences were considered statistically significant when *p* < 0.05. We found there were many duplications of individual studies included in systematic reviews and high heterogeneity existed in intervention and comparison protocols during the assessment of included reviews. We also extracted the author, published year, population type, sample size, mean difference, standard deviation, IER protocol, and CER protocol from trials of included reviews according to the inclusion criteria as prescribed in the method section to further conduct a new meta-analysis or subgroup analysis. Original studies were included in our meta-analysis, and we compared the results of our meta-analysis with those of the included systematic reviews.

### 2.6. Quality Assessment

Two authors assessed the quality of each review and RCT independently. As for included reviews, the evidence quality of included reviews was assessed by referring to the Grading of Recommendations Assessment, Development, and Evaluation (GRADE) system [29]. By using A Measurement Tool to Assess Systematic Reviews (AMSTAR 2), the methodological quality of included reviews was assessed [30]. AMSTAR 2 is composed of 16 items scored as “yes”, “no”, “partial yes”, and “no meta-analysis”. The overall quality is categorized as “high”, “moderate”, “low”, and “critically low” [30]. Reviews rated as low or critically low were not excluded to screen RCTs that met the inclusion criteria. Regarding included RCTs, the bias assessment was completed using the revised Cochrane risk-of-bias tool (ROB2), which rates five domains as being high, low, or some concerns of bias [31]. Any disagreement was resolved by discussion or the involvement of the third author.

## 3. Results

### 3.1. Results of the Search

**Figure 1:**
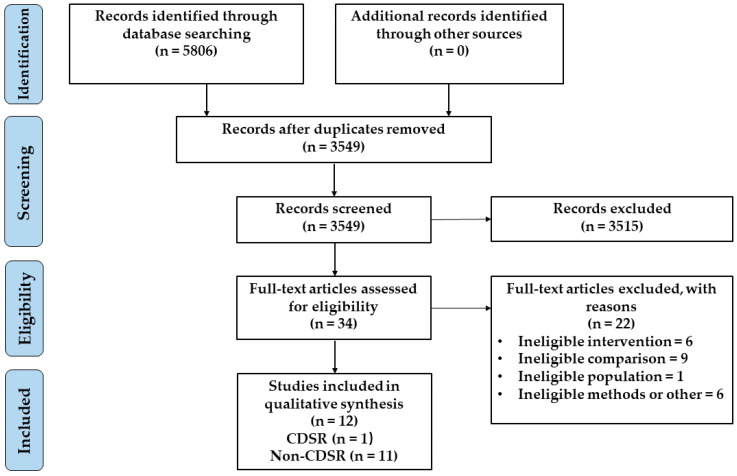
Flowchart of the study selection process.

The selection process is shown in Figure 1. A total of 5806 references were identified through database searching, and 2257 duplicate references were removed after screening. Based on the titles and abstracts, 3515 references were excluded. The remaining 34 references were then reviewed, and 22 references did not meet the inclusion criteria for different reasons. Finally, the remaining 12 reviews were included in the study, one of which was a Cochrane Database of Systematic Review (CDSR). We excluded 82 papers that were duplicated and 44 papers that did not meet the inclusion criteria from 137 trials of included reviews (Supplementary Materials S3). A total of 11 RCTs were included in our study.

### 3.2. Characteristics of Included Reviews and RCTs

The main characteristics of the included reviews and RCTs are presented in Table 1; Table 2, respectively. As shown in Table 1, only one of the twelve systematic reviews was a Cochrane review [32], and the remaining were non-Cochrane systematic reviews [19,24,25,26,33,34,35,36,37,38,39]. Most participants in the included reviews were adults with overweight or obese and were over 18 years of age. The target populations of ten reviews included adults with T2DM [19,25,26,32,34,35,36,37,38,39], of which only one review included adults with T2DM [36]. All analyzed articles of the included reviews were RCTs ranging from five to forty in number. The duration of interventions ranged from four to ninety-six weeks. The non-primary IER protocols prescribed in all included reviews differed from the duration of fasting days to the intensity of calorie restriction. Regular diet or no control were also considered as the comparison in the studies of eleven [19,24,25,26,32,33,34,35,36,38,39] and four [19,25,34,37] included reviews, respectively. One review included studies that considered the Mediterranean diet as a comparison [36], and one included VLED [34].

As shown in Table 2, no trials that performed TRF intervention met the inclusion criteria. Three RCTs used ADF [40,41,42] as IER intervention, while the others considered the 5:2 diet [20,43,44,45,46,47,48,49]. The target population of two RCTs was T2DM [43,44] and six were adults with no diabetes [20,40,41,42,48,49]. One RCT only included males [45] and two RCTs only considered females as the target population [46,47]. The duration of intervention of included RCTs that ranged from four to ninety-six weeks.

### 3.3. Outcomes for Reported Data of Included Reviews and Included RCTs

**Figure 2:**
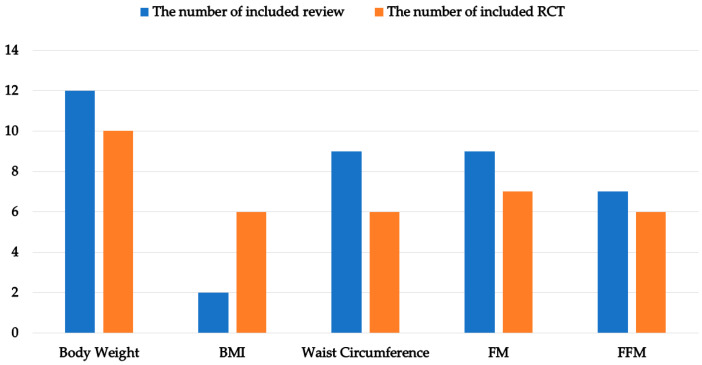
Outcomes for reported data of included reviews and included RCTs. Abbreviations: BMI, body mass index; FM, fat mass; FFM, fat free mass.

Five reported outcomes were scattered in included reviews and RCTs (Figure 2). Change in body weight was reported most frequently in included reviews, followed by waist circumference, FM, and FFM. The reported numbers of BMI between included reviews and RCTs showed an apparent difference.

### 3.4. Summary of Findings from the Meta-Analyses of the Included Reviews and Results of Our Meta-Analysis

As presented in Supplementary Materials S4, we assembled the meta-analyses data of preset outcomes from included reviews including weight loss, BMI, waist circumference, and body composition. Four separate tables were developed with the type of intervention and comparison, anticipated absolute effects (95% CI), *p*-value, and evidence quality of each outcome. Most designs of IER protocols described in RCTs of included reviews were much different from the inclusion criteria. The comparison types in most RCTs of included reviews were various including CER, regular diet, VLED, and no control. Moreover, the most of evidence quality was low or very low among outcomes of included reviews after the assessment. The limited evidence quality and heterogeneity among intervention or comparison protocols might increase bias in the conclusion. Therefore, an additional meta-analysis was developed based on the original studies from included reviews. MD (mean difference) indicates the mean difference in change between the post-intervention and baseline of the IER vs. that of the CER arms and a random-effect meta-analysis was performed. Furthermore, we conducted subgroup analysis by different IER forms (Figures S1, S4 and S5) and sensitivity analysis (Figures S2 and S3) as required. Table 3 summarizes the *p*-values of the nine quantitative reviews with meta-analyses included in the overview (the remaining three included qualitative reviews without meta-analysis were not presented in the table) and the meta-analysis we performed additionally. Among which, three quantitative reviews analyzed IER vs. CER separately from IER vs. regular diet while the remaining six did not. Moreover, the inconsistent results were explained in the discussion.

#### 3.4.1. The Effect of IER vs. CER on Body Weight

**Figure 3:**
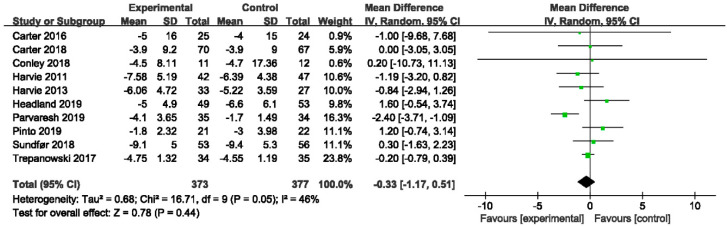
Forest plot for body weight in trials that compared IER with CER. Abbreviations: CI, confidence interval; IER, intermittent energy restriction; CER, continuous energy restriction. The unit of mean difference in body weight is kg.

In Figure 3 and Table 3, the MD in bodyweight reduction was not statistically significant in the comparison of IER and CER (MD −0.33, 95% CI −1.17 to 0.51; I^2^ = 46%) which was consistence with seven quantitative reviews [19,24,26,32,33,34,38] and three qualitative reviews [25,36,37]. However, two reviews [35,39] reported that IER could result in a greater reduction in body weight.

In the subgroup analysis, neither 5:2 diet (MD 0.19, 95% CI −0.67 to 1.05; I^2^ = 0%) or ADF (MD −1.22, 95% CI −3.37 to 0.93; I^2^ = 89%) had more effective on bodyweight that was no difference between the primary analysis (Figure S1). In the sensitivity analysis, the exclusion of Parvaresh 2019 [42] reduced the I^2^ from 46% to 0% (Figure S2).

#### 3.4.2. The Effect of IER vs. CER on BMI

**Figure 4:**
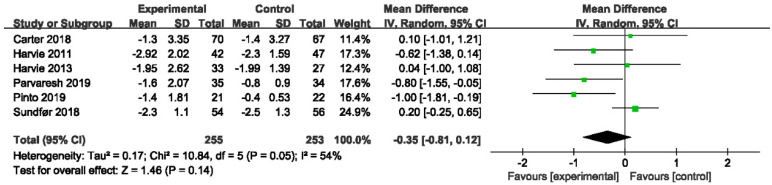
Forest plot for BMI in trials that compared IER with CER. Abbreviations: CI, confidence interval; BMI, body mass index; IER, intermittent energy restriction; CER, continuous energy restriction. The unit of mean difference in BMI is kg/m^2^.

In Figure 4, six trials of 500 participants comparing IER to CER showed that IER had no greater effective on BMI (MD −0.35, 95% CI −0.81 to 0.12; I^2^ = 54%). Inconsistent with the results of Allaf et al. [32], it reported a statistically significant effect of IER on BMI with duration ≤ 12 weeks (MD −0.43, 95% CI −0.76 to −0.10, *p* = 0.01), whereas the impact of IER disappeared when duration >12 weeks (MD −0.15, 95% CI −0.58 to 0.29, *p* = 0.51) (Table 3).

In our meta-analysis, the MD became significant after the exclusion of Sundfør 2018 [49] and the I^2^ changed from 54% to 5% (MD −0.57, 95% CI −0.97 to −0.18, I^2^ = 5%, *p* = 0.004) in the sensitivity analysis (Figure S3).

#### 3.4.3. The Effect of IER vs. CER on Waist Circumference

**Figure 5:**
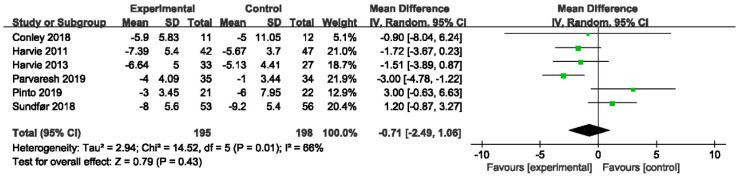
Forest plot for waist circumference in trials that compared IER with CER. Abbreviations: CI, confidence interval; IER, intermittent energy restriction; CER, continuous energy restriction. The unit of mean difference in waist circumference is cm.

In Figure 5 of our meta-analysis, trials comparing IER with CER showed no statistically significant effect regarding waist circumference (MD −0.71, 95% CI −2.49 to 1.06; I^2^ = 66%) in consistence with six quantitative reviews [19,24,26,32,35,39] and two qualitative reviews [25,36]. However, IER showed a greater reduction in waist circumference compared with CER in the results of Harris and Hamilton et al. [33] (MD −2.14, 95% CI −3.53 to −0.75, *p* = 0.002) (Table 3).

We found the heterogeneity in the subgroup analysis and sensitivity analysis could not be formally assessed in the insufficient studies with different variables among study designs.

#### 3.4.4. The Effects of IER vs. CER on Body Composition

**Figure 6:**
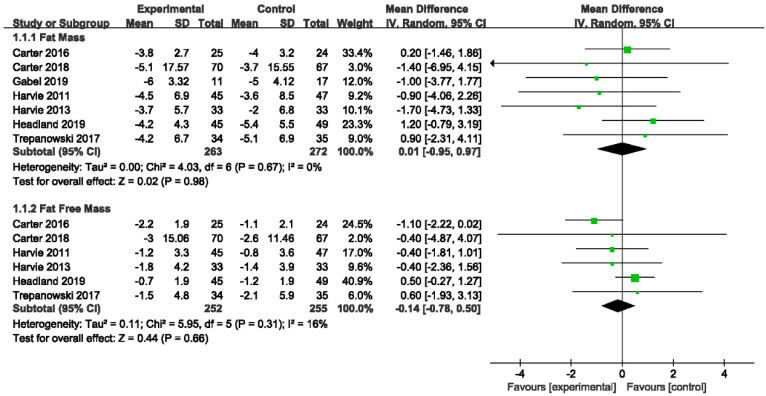
Forest plot for body composition in trials that compared IER with CER. Abbreviations: CI, confidence interval; FM, fat mass; FFM, fat-free mass; IER, intermittent energy restriction; CER, continuous energy restriction. The unit of mean difference in FM and FFM is kg.

In Figure 6 and Table 3, four quantitative reviews [19,24,26,39] and three qualitative reviews [25,36,37] showed that IER was not distinguishable from CER in effect on FM, consistent with our meta-analysis (MD −0.01, 95% CI −0.95 to 0.97; I^2^ = 0%). However, two quantitative reviews reported a statistically significant effect of IER compared with CER [33,35]. In our meta-analysis, the comparison of IER with CER suggested that IER was no more effective on FFM (MD −0.14, 95% CI −0.78 to 0.50; I^2^ = 16%), in accordance with four quantitative reviews [19,24,33,39] and two qualitative reviews [25,37]. However, the results of one review indicated that IER was associated with a greater reduction in FFM compared with CER or regular diet [26].

In the subgroup analysis, neither the 5:2 diet (MD 0.06, 95% CI −1.02 to 1.14; I^2^ = 0%) nor ADF (MD −0.19, 95% CI −2.29 to 1.91; I^2^ = 0%) had a greater effect on FM compared to CER (Figure S4). Additionally, the results of subgroup analysis revealed that neither of two subtypes (5:2 diet: MD −0.23, 95% CI −0.97 to 0.51; I^2^ = 29%; ADF: MD 0.60, 95% CI −1.93 to 3.13; *p* = 0.64) was distinguishable from CER in effect on FFM (Figure S5).

### 3.5. The Methodological Quality of the Included Reviews According to AMSTAR 2

The methodological quality assessment of the included reviews is summarized in Supplementary Materials S5. Of the 12 included reviews, one was rated as high-quality, one was of moderate-quality according to AMSTAR 2, whereas all the others were of critically low (six reviews) to low quality (four reviews). The main reason for judging six reviews to be of critically low quality according to the AMSTAR 2 was that the review authors did not carry out an adequate investigation of publication bias or discuss its likely impact on the results of the review. Regarding judging four reviews to be of low quality was that the review authors did not provide a list of the excluded studies or justify the exclusions. It is important to note that the primary studies of the included reviews were identified as a limitation of the study design that did not meet the criterion of the blinding of participants and providers to group assignments and outcome measures, many of which were categorized by reviewers as poor or moderate methodological quality and as having a high risk of bias.

### 3.6. Assessment of the Included RCTs According to ROB2

**Figure 7:**
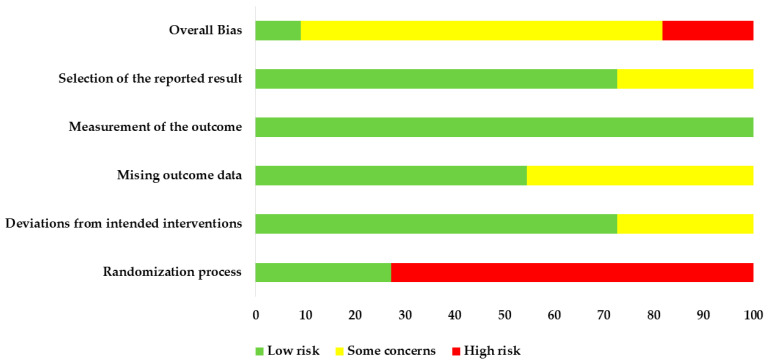
Risk of bias graph. Judgments about each risk of bias item were presented as percentages across all included studies.

**Figure 8:**
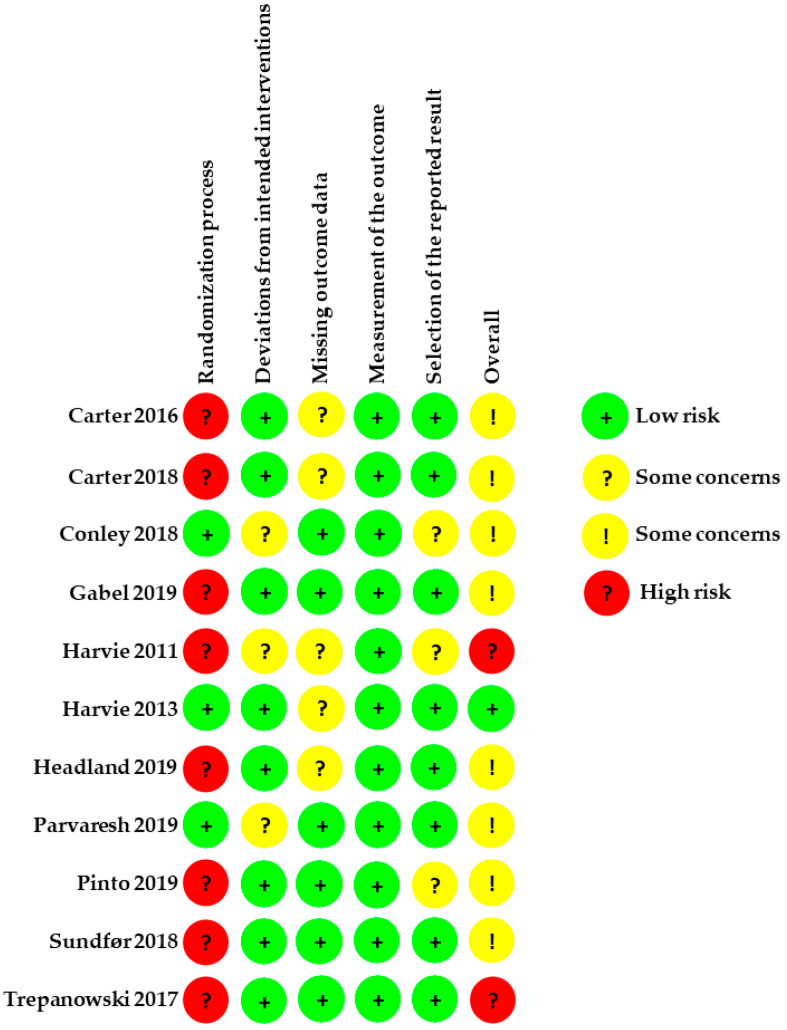
Risk of bias summary. Judgments about each risk of bias item for each included study.

The assessment results of ROB2 indicated either low or some concerns for most of the parameters of included trials (Figure 7 and Figure 8).

#### 3.6.1. Allocation

Two trials reported adequately on the randomization sequence. One stated that group allocation was established by opaque and sealed envelopes that contained the assignment for each subject [46]. The other stated that a computer-generated random number list prepared by a statistician was used [49]. Five trials stated that computer-generated random numbers were used for the assignment to either IER or CER group with equal probability [20,42,43,48,49]. Reports on the generation of the randomization sequence were unclear in the remaining 5 trials [40,41,44,45,47].

Concealment of allocation and the methods used for allocation concealment were described in 3 trials [42,45,46]. Only one RCT was reported as being double-blinded [42]. Whether the researchers of two RCTs were blinded to the intervention group was not clear [45,46]. Two trials reported that researchers and participants were not blinded to the intervention group [43,49], whereas the rest trials did not provide any information regarding blinding [20,40,41,44,47,48].

#### 3.6.2. Deviations from Intended Interventions

There was no deviation reported from the intended intervention that arose because of the experimental context in all trials. It was unclear whether an intention-to-treat analysis was carried out in three trials, thus giving some concerns about the risk of bias [42,45,47]. Intention-to-treat analysis was adequate in 8 RCTs giving a low risk of bias [20,40,41,43,44,46,48,49]. In 2 RCTs, the withdrawn participants were not included in the final analysis and consequently an intention-to-treat analysis was not applied [20,43].

#### 3.6.3. Missing Outcome Data

Four trials reported available data of outcomes for nearly all participants randomized [20,42,45,49] while the rest reported the availability of data from less than 95% of the participants [40,41,43,44,46,47,48]. There was no evidence that the result was not biased by missing the outcome data in all trials. The missingness in the outcomes of five trials could depend on its true value which reported the withdrawn reasons including poor health status [43,44,46,47,48]. As such, the 5 trials above were assessed as ‘some concerns’ in this domain. Two RCTs with missingness in the outcomes could not depend on their true value that was assessed as low risk [40,41].

#### 3.6.4. Measurement of the Outcome

There was no inappropriate method of measuring the outcome reported and no difference between intervention groups in all trials. Only one trial reported all measurements were taken by a blinded investor [45]. All RCTs in this domain were assessed as having a low risk of bias.

#### 3.6.5. Selection of the Reported Result

Three trials were assessed as ‘some concerns’ since they did not analyze the data in accordance with a prespecified analysis plan [20,45,47], whereas the rest were assessed as low risk of bias [40,41,42,43,44,46,48,49].

## 4. Discussion

### 4.1. Main Findings and Possible Explanations

This overview provides a synthesis of the state of knowledge related to the effects of IER and CER among adults with overweight or obesity based on the comparison of weight loss, BMI, waist circumference, and body composition. To integrate and sort out the consistent and different parts for improving the integrity and accuracy of the evidence. We conducted an additional meta-analysis including original trials from included reviews that met the inclusion criteria and compared the results with those of the included systematic reviews and meta-analyses. In addition, we also assessed RCTs by using ROB2. Although we found numerous limitations of the current evidence, the results of the original trials did not show any significant differences in the comparison of IER and CER for anthropometric outcomes.

There were some inconsistencies between the results of our meta-analysis and included reviews among outcomes. As for body weight, Schwingshackl et al. [35] and He et al. [39] reported a greater reduction of 0.55 and 0.95 kg, respectively, in the IER group, while the study designs of some trials were not completely accordant with inclusion criteria. For instance, the intervention of one study combined exercise with IER [50]. One trial combined two consecutive days with 70% energy restriction and 5 days on the Mediterranean diet as IER intervention [51], while another trial changed the intensity of IER or fasting days per week that were much different from our inclusion criteria [52]. The influence of other different interventions might increase the effect of IER on weight loss such as combination with physical activity and harder intensity of calorie restriction. Regarding the reduction of BMI, Allaf et al. [32] conducted meta-analyses according to the length of duration. The results indicated that IER could result in more reduction in BMI in the short term, while the advantage disappeared when the duration was more than 12 weeks. In our meta-analysis, the duration of trials was almost more than 12 weeks, and the results also showed no significant difference between the two interventions. As for waist circumference, although Harris and Hamilton et al. [33] reported that IER was associated with more reduction in waist circumference, the number of participants was unclear in RCTs. On the other hand, the heterogeneity during meta-analysis in this study could not be assessed formally since insufficient studies with various results. Thus, the effect of IER on waist circumference needs more studies. With regard to FM, Harris and Hamilton et al. [33] and Schwingshackl et al. [35] reported that IER was associated with more reduction in FM. The possible reasons for the difference were discussed above. As for FFM, one study [53] in Roman et al. [26] might contribute to a greater effect of IER on FFM since its change in intensity of calorie restriction and duration of fasting days results in inconsistency.

It is worth noting that heterogeneity exists since the IER described in some trials did not meet the inclusion criteria, for example, more than two consecutive fasting days per week [54], the period unit of diet protocol is rather than one week [53,54,55] or combined physical activity [56,57]. Moreover, various conditions of the target subject, such as the age, gender, or BMI range might contribute to the discrepancies in the results: For instance, participants with normal weight were also included in two included reviews [25,32], and only T2DM or male or female were included in some trials [43,44,45,46,47]. Furthermore, the distinguishing discussion between adults with overweight and those with obesity was not clear in the analysis of included trials.

The quality of evidence was found to be very low to moderate variously among different outcomes according to GRADE which was associated with risk of bias and small sample size in original trials. The concealment of both researchers and participants in most trials of included reviews was not available that increases the risk of bias. It is worth noting that whether the included reviews compared IER with CER separately from IER vs. regular diet or not, some IER protocols with various designs in included reviews were not primary subtypes, which were not included in our meta-analysis might contribute to the inconsistency or consistency between results. Overall, the low to critically very low certainty of included evidence prevents us from drawing any firm conclusions regarding the effectiveness of all IER protocols compared to CER, which all require further study.

There are several other research directions for the future. First, there is limited convincing evidence related to the effect of CR on adults with normal weight which we found in some trials of the included reviews. More studies are needed to find if there are similar benefits of CR when interpreting to different populations. Second, for the target subjects, the range of age, BMI, and gender are important elements regarding the basic metabolism in the study design, which might be related to the discrepancy in outcomes. Third, the other forms of IER are not considered to compare with CER, and more studies of high quality are needed for exploring the advantages and benefits of non-primary or new IER protocols. Fourth, the flexibility of diet strategies in IER or CER and the difference between the daily-life trajectory of a single person should be considered. It could be more individualized and specific for each adult with overweight or obesity, since in the context of increasing adults with overweight and obesity around the world, to adhere IER or CER for the long term may be an efficient and economical approach to maintaining a long and healthy life [58,59].

### 4.2. Limitations of the Present Study

Some limitations need to be acknowledged in the present work. First, we have less confidence in the accuracy of the compared results since the intervention designs of trials in included reviews are much different from the 5:2 diet and ADF. Furthermore, the small number of trials included in our meta-analysis might be insufficient to enhance the evidence. Second, all trials of our meta-analysis were the 5:2 diet and ADF with similar intensity of fasting days whereas no TRF vs. CER meeting the inclusion criteria was found during study selection, which may decrease the accuracy of the results. On the other hand, we did not analyze studies that conducted non-CR diet regimens that were excluded during the selection process. Third, considering the insufficient original trials that met the inclusion and the risk of bias, the subgroup analysis of only T2DM or male or female was not implemented in our meta-analysis. Fourth, we did not explore the influence on adherence, appetite, or adverse events of IER and CER intervention and their association with follow-up time. Therefore, it is worthy of more studies in the future. Fifth, at present, there is limited evidence focused specifically on adults with T2DM, and as such it is unclear whether two interventions would have the same results reported above in this work when delivered to adults with T2DM. Sixth, a critical limitation with currently available evidence is the poor quality of reviews that suggests a need to improve the conducting and reporting of systematic reviews.

## 5. Conclusions

Although the results of this work showed no difference between IER and CER for anthropometric outcomes, the evidence obtained in the present work confirmed that IER and CER have an essential and active influence on weight loss among adults with overweight or obesity for a short term in their life. However, researchers should strive to design and conduct new long-term RCT studies that help to improve the evidence quality of IER protocols implemented in adults with overweight or obesity and adults with diabetes.

## Figures and Tables

**Table 1:** Characteristics of included reviews.

Author, Year	Date of Search	No. Studies Included	Population				Intervention		Comparison Intervention	
			Type	Sample Size	Age (Year)	BMI (kg/m^2^)	Diet Form	Total Duration (Week)	Diet Form	Total Duration (Week)
Cochrane systematic review										
Allaf, 2021 [32]	2019.12	26	1. Male 2. Menopausal women with metabolic syndrome 3. Adults with T2DM 4. Professional cyclists 5. Adults with overweight or obesity	1125	18–70	20–45	IER: 5:2 diet/ADF/TRF/Other^*^	16–96	CER/Regular diet	4–96
Non-Cochrane systematic review										
Seimon, 2015 [25]	2014.11	12	1. Male with T2DM overweight/obesity 2. Female with overweight or obesity 3. Male with obesity and female 4. Male with obesity and female with T2DM	1440	17–79	20–45	IER: ADF/Other^*^	5–50	CER/Regular diet/No control	5–50
Davis, 2016 [37]	2013.09	8	1. Adults with T2DM 2. Postmenopausal women 3. Premenopausal women	390	34.3–61.8	28.6–37.3	IER: 5:2 diet/Other^*^	5–48	CER/No control	5–48
Headland, 2016 [34]	2016.04	9	1. All Female 2. All Male 3. All female and male 4. Female with T2DM	981	18–70	24–40	IER: 5:2 diet/Other^*^	10–96	CER/Regular diet/VLED/No control	10–96
Cioffi, 2018 [19]	2018.05	11	All adults with overweight/obesity (1) Men and women (2) All men (3) Adults with T2DM (4) Dysmetabolic conditions (5) All women	630	30–71	24–46	IER: 5:2 diet/ADF/Other^*^	8–24	CER/Regular diet/No control	8–24
Harris and Hamilton, 2018 [33]	2015.11	6	Adults with overweight or obesity, except adults with diabetes	400	37–49	26–35.6	IER: 5:2 diet/ADF/Other^*^	12–48	CER/Regular diet	12–48
Harris and McGarty, 2018 [38]	2015.09	5	All adults with overweight/obesity (1) Men and women (2) Adults with T2DM (3) All women	376	42.6–61.0	33.1–44.6	IER: Other^*^	14–48	CER/Regular diet	14–48
Roman, 2019 [26]	2018.02	9	1. All adults with overweight/obesity 2. Only adults with diabetes 3. Excluded adults with diabetes	782	39.6–61.5	24–45	IER: 5:2 diet/Other^*^	12–52	CER/Regular diet	12–52
Vitale, 2020 [36]	2020.01	5	Adults with T2DM and obesity (had T2DM between 1 and 25 years in duration)	351	46–71	27.6–41.8	IER: 5:2 diet/Other^*^	12–24	CER/Mediterranean diet/	12–24
Guerrero, 2021 [24]	2019.01	18	Adults with overweight/obesity	1219	18–70	≥25	IER: 5:2 diet/ADF/Other^*^	6–48	CER/Regular diet	6–48
He, 2021 [39]	2019.12	11	All adults with overweight/obesity (1) Men and women (2) Adults with T2DM (3) All women (4) All men	850	28–71	26–43	IER: 5:2 diet/ADF/Other^*^	8–48	CER/Regular diet	8–48
Schwingshackl, 2021 [35]	2019.03	17	1. Adults with overweight/obesity 2. Adults with T2DM	1328	31.7–67.6	26–35.3	IER: 5:2 diet/ADF/Other^*^	12–52	CER/Regular diet	12–52

Abbreviations: ADF, alternate day fasting; BMI, body mass index; CER, continuous energy restriction; ER, energy restriction; IER, intermittent energy restriction; N/A: not applicable; PF, periodic fasting; TRF, time-restricted feeding; T2DM, diabetes mellitus type 2; VLED, very low energy diet. No IER protocols are prescribed in the inclusion criteria.

**Table 2:** Characteristics of included RCTs.

Author, Year	Population				Intervention		Comparison	Duration (Week)
	Type	Sample Size	BMI (kg/m^2^)	Age (Year)	IER Form	IER Protocol	CER Protocol	
Carter, 2016 [43]	T2DM	EG:25 CG:24	≥27	>18	5:2 Diet	400–600 kcal/day on 2 fast days and regular diet on 5 feed days	1200–1500 kcal/day	12
Carter, 2018 [44]	T2DM	EG:70 CG:67	≥27	>18	5:2 Diet	500–600 kcal/d for 2 days and regular diet for 5 days	1200–1500 kcal/day	96
Conley, 2018 [45]	Male with no T2DM	EG:11 CG:12	≥30	55–75	5:2 Diet	600 kcal/day on 2 fast days and regular diet on 5 feed days	25% energy restriction every day	24
Gabel, 2019 [41]	Adults with no T2DM	EG:11 CG:17	25.0–39.9	18–65	ADF	25% of the energy need on fast days and 125% of energy needs on feed days	75% energy needs every day	48
Harvie, 2011 [47]	Premenopausal women with no T2DM	EG:45 CG:47	24–40	30–45	5:2 Diet	25% of the energy need on 2 fast days and regular diet for 5 days	25% energy restriction every day	24
Harvie, 2013 [46]	Women with no T2DM	EG:33 CG:33	24–45	20–69	5:2 Diet	25% of the energy need on 2 fast days and regular diet for 5 days	25% energy restriction every day	16
Headland, 2019 [48]	Adults with no T2DM	EG:49 CG:53	>25	18–72	5:2 Diet	500/600 kcal (F/M) on 2 fast days and regular diet for 5 days	1000–1200 kcal/day (F/M)	48
Parvaresh, 2019 [42]	Adults with no T2DM	EG:35 CG:34	25–40	25–60	ADF	25% energy needs on fast days; 100% needs on alternating feast days	25% energy restriction every day	8
Pinto, 2019 [20]	Adults with no T2DM	EG:21 CG:22	>25	35–75	5:2 Diet	600 kcal on 2 fast days and regular diet for 5 days	25% energy restriction every day	4
Sundfør, 2018 [49]	Adults with no T2DM	EG:54 CG:58	30–45	21–70	5:2 Diet	400/600 kcal (F/M) on 2 fast days and regular diet for 5 days	26–28% energy restriction every day	48
Trepanowski, 2017 [40]	Adults with no T2DM	EG:34 CG:35	25–39.9	18–64	ADF	25% of the energy need on fast days and 125% of energy needs on feed days	25% energy restriction every day	24

Abbreviations: BMI, body mass index; IER, intermittent energy restriction; CER, continuous energy restriction; T2DM, diabetes mellitus type 2; EG, experimental group; CG, control group.

**Table 3:** Summarizes the *p*-values of included reviews with meta-analysis and our meta-analysis.

Author, Year	Body Weight	BMI	Waist Circumference	FM	FFM
IER vs. CER (Analyzed separately from IER vs. regular diet)					
Harris and Hamilton, 2018	0.156 ^b^	/	0.002 ^b^	0.014 ^c^	0.958 ^b^
Allaf, 2021 (≤12 weeks)	0.05 ^b^	0.01 ^a^	0.20 ^a^	/	/
Allaf, 2021 (>12 weeks)	0.33 ^b^	0.51 ^b^	0.49 ^c^	/	/
Schwingshackl, 2021	0.02 ^a^	/	0.25 ^a^	0.007 ^a^	/
IER vs. CER (Not analyzed separately from IER vs. regular diet)					
Headland, 2016	0.458 ^c^	/	/	/	/
Cioffi, 2018	0.27 ^a^	/	0.83 ^c^	0.66 ^a^	0.58 ^a^
Harris and McGarty, 2018	0.15 ^c^	/	/	/	/
Roman, 2019	0.29 ^a^	/	0.71 ^b^	0.56 ^b^	0.03 ^b^
Guerrero, 2021	N ^a^	N ^a^	N ^b^	N ^c^	N ^c^
He, 2021	0.006 ^a^	/	0.61 ^b^	0.08 ^a^	0.09 ^a^
Our meta-analysis	0.44	0.14	0.43	0.98	0.66

Abbreviations: BMI, Body mass index, CER, Continuous energy restriction; FM: Fat mass; FFM: Fat-free mass; IER, Intermittent energy restriction. *p*-value ^a^: The evidence quality was identified as moderate. *p*-value ^b^: The evidence quality was identified as low. *p*-value ^c^: The evidence quality was identified as very low. N: *p*-value was not available.

## Data Availability

Not applicable.

## Supplementary Materials

The following supporting information can be downloaded at: https://www.mdpi.com/article/10.3390/nu14112315/s1, Figure S1: Forest plot for body weight in trials that compared subtypes of IER with CER, Figure S2: Forest plot of sensitivity analysis for body weight in trials that compared IER with CER, Figure S3: Forest plot of sensitivity analysis for BMI in trials that compared IER with CER, Figure S4: Forest plot for FM in trials that compared subtypes of IER with CER, Figure S5: Forest plot for FFM in trials that compared subtypes of IER with CER, Supplementary Materials S1: PRISMA-2009-checklist, Supplementary Materials S2: Search strategy, Supplementary Materials S3: Information of excluded RCTs. Supplementary Materials S4: Summary of findings from the meta-analysis of the outcomes, Supplementary Materials S5: Quality assessment of systematic reviews by AMSTAR 2.

## Abbreviations

ADF	Alternate Day Fasting
AMSTAR	A Measurement Tool to Assess Systematic Reviews
BMI	Body Mass Index
CER	Continuous Energy Restriction
CR	Calorie Restriction
CVD	Cardiovascular Disease
CG	Control Group
CI	Confidence Interval
ER	Energy Restriction
EG	Experimental Group
FM	Fat Mass
FFM	Fat-Free Mass
GRADE	The Grading of Recommendations Assessment, Development and Evaluation
IER	Intermittent Energy Restriction
MD	Mean Difference
PRISMA	The Statement of Preferred Reporting Items for Systematic Review and Meta-analyses
PF	Periodic Fasting
RCT	Randomized Control Trial
ROB	The Cochrane Risk-of-Bias Tool
TRF	Time-Restricted Feeding
T2DM	Diabetes Mellitus Type 2
VLED	Very Low Energy Diet
WHO	World Health Organization
