# Supplementary material for: Comparison of the Effects of Intermittent Energy Restriction and Continuous Energy Restriction among Adults with Overweight or Obesity: An Overview of Systematic Reviews and Meta-Analyses
Source: Nutrients. 2022 May 31;14(11):2315. doi: 10.3390/nu14112315 (PMC9183159; doi:10.3390/nu14112315)
Supplement: Supplementary file 1 [file nutrients-14-02315-s001.zip › Supplementary Materials S2. Search Strategy.pdf]

Supplementary Materials S2. Search Strategy

The Cochrane Library of Systematic Review

#1 overweight

#2 obesity

#3 obese

#4 fat

#5 {OR #1-#4}

#6 "intermittent energy restriction"

#7 "intermittent calorie restriction"

#8 "intermittent fasting"

#9 "fasting"

#10 "alternate-day fasting"

#11 "alternate day fasting"

#12 "5:2 diet"

#13 "time-restricted feeding"

#14 "time restricted feeding"

#15 "time-restricted eating"

#16 "time restricted eating"

#17 "time-restricted diet"

#18 "time restricted diet"

#19 {OR #6-#18}

#20 "continuous energy restriction"

#21 "continuous calorie restriction"

#22 "daily energy restriction"

#23 "daily calorie restriction"

#24 "calorie restriction"

#25 {OR #20-#24}

#26 "meta-analysis"

#27 "meta-analyses"

#28 "meta analy*"

#29 "metanaly*"

#30 "metaanaly*"

#31 "met analy*"

#32 {OR #26-#31}

#33 "systematic reviews"

#34 "systematic review"

#35 "system review"

#36 {OR #33-#35}

#37 #5 and #19

#38 #5 and #25

#39 #37 or #38

#40 #32 or #36

#41 #39 and #40

Clinical Trials

1. “overweight” [Mesh]
2. “obesity” [Mesh]
3. “obese” [Mesh]
4. (“meta-analysis” [Mesh]) OR (“systematic review” [Mesh])

PubMed

1. “overweight” [Mesh]
2. “obesity” [Mesh]
3. “obese” [Mesh]
4. (“overweight” [All fields]) OR (“obesity” [All fields]) OR (“obese” [All fields]) OR (“fat” [All fields])
5. OR/1-4
6. (“intermittent energy restriction” [All fields]) OR (“intermittent calorie restriction” [All fields]) OR (“intermittent fasting” [All fields]) OR (“fasting” [All fields]) OR (“alternate-day fasting” [All fields]) OR (“alternate day fasting” [All fields]) OR (“5:2 diet” [All fields]) OR (“time-restricted feeding” [All fields]) OR (“time restricted feeding” [All fields]) OR (“time-restricted eating” [All fields]) OR (“time restricted eating” [All fields]) OR (“time-restricted diet” [All fields]) OR (“time restricted diet” [All fields])
7. (“continuous energy restriction” [All fields]) OR (“continuous calorie restriction” [All fields]) OR (“daily energy restriction” [All fields]) OR (“daily calorie

restriction” [All fields]) OR (“calorie restriction” [All fields])

8. (“systematic review” [Publication Type]) OR (“meta-analysis” [Publication Type])
9. (“meta-analysis” [All fields]) OR (“meta-analyses” [All fields]) OR (“meta analy*” [All fields]) OR (“metanaly*” [All fields]) OR (“metaanaly*” [All fields]) OR (“met analy*” [All fields])
10. (“systematic reviews” [All fields]) OR (“systematic review” [All fields]) OR (“system review” [All fields])
11. AND/5,6
12. AND/5,7
13. OR/11,12
14. OR/8-10
15. AND/13,14

Medline (Ovid)

1. (overweight or obesity or obese or fat) af.
2. (intermittent energy restriction or intermittent calorie restriction or intermittent fasting or fasting or alternate-day fasting or alternate day fasting or 5:2 diet or time-restricted feeding or time restricted feeding or time-restricted eating or time restricted eating or time-restricted diet or time restricted diet) af.
3. (continuous energy restriction or continuous calorie restriction or daily energy restriction or daily calorie restriction or calorie restriction) af.
4. (systematic review or meta-analysis). pt.

5. (meta-analysis or meta-analyses or meta analy* or metanaly* or metaanaly* or met analy*) af.
6. (systematic reviews or systematic review or system review) af.

Embase (Ovid)

1. (overweight or obesity or obese or fat) af.
2. (intermittent energy restriction or intermittent calorie restriction or intermittent fasting or fasting or alternate-day fasting or alternate day fasting or 5:2 diet or time-restricted feeding or time restricted feeding or time-restricted eating or time restricted eating or time-restricted diet or time restricted diet) af.
3. (continuous energy restriction or continuous calorie restriction or daily energy restriction or daily calorie restriction or calorie restriction) af.
4. (systematic review or meta-analysis). pt.
5. (meta-analysis or meta-analyses or meta analy* or metanaly* or metaanaly* or met analy*) af.
6. (systematic reviews or systematic review or system review) af.

Scopus

(ALL (overweight) OR ALL (obesity) OR ALL (obese) OR ALL (fat)) AND (ALL (intermittent) AND ALL (energy) AND ALL (calorie) AND ALL (restriction) AND ALL (fasting) AND ALL (alternate-day) AND ALL (alternate) AND ALL (day) AND ALL (5:2) AND ALL

(diet) AND ALL (time-restricted) AND ALL (feeding) AND ALL (eating) AND ALL (diet)) OR (ALL (overweight) OR ALL (obesity) OR ALL (obese) OR ALL (fat)) AND (ALL (continuous) AND ALL (energy) AND ALL (calorie) AND ALL (restriction) AND ALL (daily)) AND (ALL (meta) AND ALL (analysis) AND ALL (analyses) AND ALL (systematic) AND ALL (review) AND ALL (reviews))

Web of Science

((("overweight" OR "obesity" OR "obese" OR "fat" (Topic)) AND ("intermittent energy restriction" OR "intermittent calorie restriction" OR "intermittent fasting" OR "fasting" OR "alternate-day fasting" OR "alternate day fasting" OR "5:2 diet" OR "time-restricted feeding" OR "time restricted feeding" OR "time-restricted eating" OR "time restricted eating" OR "time-restricted diet" OR "time restricted diet" (Topic))) OR (("overweight" OR "obesity" OR "obese" OR "fat" (Topic)) AND ("continuous energy restriction" OR "continuous calorie restriction" OR "daily energy restriction" OR "daily calorie restriction" OR "calorie restriction" (Topic)))) AND ("meta-analysis" OR "meta-analyses" OR "meta analy*" OR "metanaly*" OR "metaanaly*" OR "met analy*" OR "systematic reviews" OR "systematic review" OR "system review" (Topic))
