# Supplementary material for: Comparison of the Effects of Intermittent Energy Restriction and Continuous Energy Restriction among Adults with Overweight or Obesity: An Overview of Systematic Reviews and Meta-Analyses
Source: Nutrients. 2022 May 31;14(11):2315. doi: 10.3390/nu14112315 (PMC9183159; doi:10.3390/nu14112315)
Supplement: Supplementary file 1 [file nutrients-14-02315-s001.zip › Supplementary Materials S3. Information of excluded RCTs.pdf]

Supplementary Materials S3. Information of excluded RCTs

Author, year	Title	Intervention	Comparison	Reason for exclusion
Heilbronn 2006	Effect of 6-Month Calorie Restriction on Biomarkers of Longevity, Metabolic Adaptation, and Oxidative Stress in Overweight Individuals A Randomized Controlled Trial	CR with exercise	Weight maintenance diet	Neither intervention nor comparison meets the inclusion criteria.
Cho 2019	Efects of alternate day fasting and exercise on cholesterol metabolism in overweight or obese adults: a pilot randomized controlled trial.	● ADF and exercise (E-ADF);<br>● ADF;<br>● Exercise	Ad libitum	The Comparison is not CER.
Chow 2019	Time restricted eating (TRE) promotes weight loss, alters on basal metabolism, maximal strength, body composition, inflammation, and cardiovascular risk	TRF	Ad libitum	The Comparison is not CER.
Brehm 2003	A Randomized Trial Comparing a Very Low Carbohydrate Diet and a Calorie-Restricted Low Fat Diet on Body Weight and Cardiovascular Risk Factors in Healthy Women	Very low carbohydrate diet	Calorie-restricted low-fat diet	Neither intervention nor comparison meets the inclusion criteria.
Kroeger 2018	Eating behavior traits of successful weight losers during 12 months of alternate-day fasting: An exploratory analysis of a randomized controlled trial	ADF	CER	At half of the intervention, it changed the CER to ad libitum in the comparison group.
Ash 2003	Effect of intensive dietetic interventions on weight and glycaemic control in overweight men with Type II diabetes: a randomised trial	IF: Fasting for 4 consecutive days each week	CER	The intervention is not the primary IER protocol.
Arguin 2012	Short- and long-term effects of continuous versus intermittent restrictive diet approaches on body composition and the metabolic profile in overweight and obese postmenopausal women: a pilot study	IF: 5-week energy restriction periods were followed by 5-week weight stabilization periods	CER: 15 weeks of energy restriction was followed by 5 weeks of weight stabilization.	The intervention is not the primary IER protocol.
Amodio 2016	Time restricted feeding (TRF) enhances weight loss eficiency in dietary restricted women with metabolic syndrome.	TRF	Ad libitum	The Comparison is not CER.
Bhutani 2013	Alternate day fasting and endurance exercise combine to reduce body weight and favorably alter plasma lipids in obese humans.	● ADF plus endurance exercise;<br>● ADF;	Ad libitum	The Comparison is not CER.

		● Exercise		
Byrne 2018	Intermittent energy restriction improves weight loss efficiency in obese men: the MATADOR study	IF: Fasting for four non-consecutive periods of two weeks	CER	The intervention is not the primary IER protocol.
Corley 2019	Changes in resting energy expenditure with intermittent fasting versus continuous daily restriction-a randomised controlled trial.	5:2 diet	CER	Incomplete article.
Coutinho 2017	Compensatory mechanisms activated with intermittent energy restriction: a randomized control trial.	IF: 3 non-consecutive days of partial fasting per week	CER	The intervention is not the primary IER protocol.
De Groot 1989	Adaptation of energy metabolism of overweight women to alternating and continuous low energy intake.	IER	/	The study design is non-RCT.
Ferraris 2019	Time restricted feeding in high-level athletes: a pilot study	TRF	Ad libitum	The comparison is not CER.
Beaulieu 2019	Matched Weight Loss Through Intermittent or Continuous Energy Restriction Does Not Lead To Compensatory Increases in Appetite and Eating Behavior in a Randomized Controlled Trial in Women with Overweight and Obesity	IF： To consume a very low-calorie diet (75% energy restriction) during the 3 fast days (Saturday, Monday, Wednesday) per week	CER	The intervention is not the primary IER protocol.
Keogh 2014	Effects of intermittent compared to continuous energy restriction on short-term weight loss and long-term weight loss maintenance.	IF: CR for 1 week, followed by 1 week of their usual diet	CER	The intervention is not the primary IER protocol.
Hutchison 2019	Effects of intermittent versus continuous energy intakes on insulin sensitivity and metabolic risk in women with overweight.	IF: Fasting for 3 non-consecutive days/week	CER	The intervention is not the primary IER protocol.
Molina 2019	The Mediterranean diet pattern with intermittent semi-fasting may facilitate weight loss: randomised controlled trial.	Mediterranean diet + semi-fast	Standard hypocaloric Mediterranean diet	Neither intervention nor comparison meets the inclusion criteria.
Hill 1989	Evaluation of an alternating calorie diet with and without exercise in the treatment of obesity.	ADF with behavior therapy	CR with behavior therapy	Neither intervention nor comparison meets the inclusion criteria.
Mraovic 2018	The effects of different caloric restriction diets on anthropometric and cardiometabolic risk factors in overweight and obese females.	CR alternating daily diets: one day 70% and the following day 30% caloric restriction	CER	The intervention is not the primary IER protocol.

Panizza 2019	Effects of intermittent energy restriction combined with a Mediterranean diet on reducing visceral adiposity: A randomized active comparator pilot study.	IER+MED diet	DASH diet	Neither intervention nor comparison meets the inclusion criteria.
Li 2017	Effects of A one-week fasting therapy in patients with type-2 diabetes mellitus and metabolic syndrome—a randomized controlled explorative study.	IF: 7 modified fasting days every 4 months period	Mediterranean diet	Neither intervention nor comparison meets the inclusion criteria.
Kahleova 2014	Eating two larger meals a day (breakfast and lunch) is more effective than six smaller meals in a reduced-energy regimen for patients with type 2 diabetes: A randomised crossover study.	The A6 regimen: consisted of three main meals (breakfast, lunch and dinner), and three smaller snacks in between	The B2 regimen consisted of breakfast (eaten between 06:00 and 10:00 hours) and lunch (eaten between 12:00 and 16:00 hours)	The study design is non-RCT.<br>Neither intervention nor comparison meets the inclusion criteria.
Lantz 2003	Intermittent versus on-demand use of a very low-calorie diet: A randomized 2-year clinical trial	Very-low-calorie diets for 2 weeks every third month	Very-low-calorie diets whenever their body weight passed an individualized cut-off level	Neither intervention nor comparison meets the inclusion criteria.
Huffman 2012	Caloric Restriction Alters the Metabolic Response to a Mixed-Meal: Results from a Randomized, Controlled Trial	CR and exercise	CER	The intervention combined with physical activity.
Moro 2016	Effects of eight weeks of time-restricted feeding (16/8) on basal metabolism, maximal strength, body composition, inflammation, and cardiovascular risk factors in resistance-trained males	TRF	Ad libitum	The Comparison is not CER.
Rossner 1998	Intermittent vs continuous VLCD therapy in obesity treatment	Intermittent very-low-calorie diet: fasting for three non-consecutive periods of two weeks	Continuous very-low-calorie diet	The intervention is not the primary IER protocol.
Klempel 2012	Intermittent fasting combined with calorie restriction is effective for weight loss and cardio-protection in obese women	IF: severe restriction 1 d per week	Ad libitum	Neither intervention nor comparison meets the inclusion criteria.
Stekovic 2019	Alternate day fasting improves physiological and molecular markers of aging in healthy, non-obese humans.	ADF	Ad libitum	The study design is non-RCT.<br>The Comparison is not CER.<br>The subjects are non-obese.
Teng 2011	Efficacy of fasting calorie restriction on quality of life among aging men.	Fasting calorie restriction (FCR)	Ad libitum	Neither intervention nor comparison meets the inclusion criteria.

		dietary regime: two days of Muslim Sunnah fasting combined with a reduction of 300–500 kcal/d from the habitual energy intake		
Teng 2013	Improvement of metabolic parameters in healthy older adult men following a fasting calorie restriction intervention.	Fasting calorie restriction (FCR) dietary regime: two days of Muslim Sunnah fasting combined with a reduction of 300–500 kcal/d from the habitual energy intake	Ad libitum	Neither intervention nor comparison meets the inclusion criteria.
Todd 2015	Weight loss intervention trial comparing intermittent low carbohydrates versus continuous Mediterranean diet.	Intermittent low-energy diets (ILEDs): 2 days of Optifast and 5 days of a Mediterranean diet/week	Continuous low-energy diets (CLEDs)	The intervention is not the primary IER protocol.
Johnson 2007	Alternate Day Calorie Restriction Improves Clinical Findings and Reduces Markers of Oxidative Stress and Inflammation in Overweight Adults with Moderate Asthma	ADF	/	The study design is non-RCT.<br>The comparison was not mentioned in the study design.
Tinsley 2017	Time-restricted feeding in young men performing resistance training: a randomized controlled trial.	TRF with resistance training	Normal diet with resistance training	Interventions of both groups are combined with physical activity.
Tinsley 2019	Time-restricted feeding plus resistance training in active females: a randomized trial.	TRF with resistance training	Normal diet with resistance training	Interventions of both groups are combined with physical activity.
Varady 2011	Comparison of effects of diet versus exercise weight loss regimens on LDL and HDL particle size in obese adults.	ADF	● CER;<br>● Exercise;<br>● Ad libitum	Not provide the mean difference on change from baseline of the IER vs the CER arms.
Varady 2013	Alternate day fasting for weight loss in normal weight and overweight subjects: a randomized controlled trial.	ADF	Ad libitum	The Comparison is not CER.
Michalsen 2005	Mediterranean diet or extended fasting's influence on changing the intestinal	IF: 4 fast days of 85% CR alternated	Mediterranean diet	Neither intervention nor comparison meets the inclusion criteria.

	microflora, immunoglobulin A secretion and clinical outcome in patients with rheumatoid arthritis and fibromyalgia: an observational study	with 4 feed days of 25% CR		
Williams 1998	The effect of short periods of caloric restriction on weight loss and glycemic control in type 2 diabetes	Very-low-calorie diet for 5 consecutive days every 5 weeks	Very-low-calorie diet for 1 day/week	Neither intervention nor comparison meets the inclusion criteria.
Hussin 2013	Efficacy of fasting and calorie restriction (FCR) on mood and depression among ageing men	Reduction of 300 to 500 kcal/day from participants baseline energy intake combined with two days of Muslim Sunnah fasting per week	Ad libitum	Neither intervention nor comparison meets the inclusion criteria.
Heilbronn 2005	Alternate-day fasting in nonobese subjects: effects on body weight, body composition, and energy metabolism	ADF	/	The study design is non-RCT.<br>The comparison was not mentioned in the study design.
Wing 1994	Year-long weight loss treatment for obese patients with type II diabetes: does including an intermittent very-low-calorie diet improve outcome?	A very low-calorie diet of 400 to 500 kcal per day alternating with the balanced low-calorie diet	A balanced low-calorie diet of 1,000 to 1,000 kilocalories (kcal) per day	The intervention is not the primary IER protocol.
Wing 2003	Prescribed “breaks” as a means to disrupt weight control efforts.	To take a 6-week break after the seventh behavioral lesson.	To take 2-week breaks after the third, sixth, and ninth behavioral lessons.	Neither intervention nor comparison meets the inclusion criteria.
Viegener 1990	Effects of an intermittent, low-fat, low-calorie diet in the behavioral treatment of obesity.	Behavior therapy plus an intermittent, low-fat, 800-calorie per day diet, used 4 days per week	Behavior therapy plus a continuous 1200-calorie per day	Neither intervention nor comparison meets the inclusion criteria.
