# Supplementary material for: Comparison of the Effects of Intermittent Energy Restriction and Continuous Energy Restriction among Adults with Overweight or Obesity: An Overview of Systematic Reviews and Meta-Analyses
Source: Nutrients. 2022 May 31;14(11):2315. doi: 10.3390/nu14112315 (PMC9183159; doi:10.3390/nu14112315)
Supplement: Supplementary file 1 [file nutrients-14-02315-s001.zip › Supplementary Materials S4. Summary of findings from the meta-analysis of the outcomes.pdf]

Supplementary Materials S4. Summary of findings from the meta-analysis of the outcomes

Summary of findings from the meta-analysis for the outcome of body weight (kg)

Systematic reviews (First author, year)	Outcome	Number of participants (studies)	Type of intervention	Type of comparison	Anticipated absolute effects (95%CI), P	Quality of evidence (GRADE)
Headland, 2016	Body weight	433 (7RCTs)	5:2 diet/Other*	CER/Regular diet/VLED/No control	MD=0.084 (-0.139, 0.308), P=0.458	Very low quality
Cioffi, 2018		666 (10RCTs)	5:2 diet/ADF/Other*	CER/Regular diet/No control	WMD=-0.61 (-1.70, 0.47), P=0.27	Moderate quality
Harris and Hamilton, 2018		287 (4RCTs)	5:2 diet/ADF/Other*	CER	WMD=-1.03 (-2.46, 0.40), P=0.156	Low quality
		62 (2RCTs)	5:2 diet/ADF/Other*	Regular diet	WMD=-4.14 (-6.30, -1.99), P<0.001	Very low quality
Harris and McGarty, 2018		317 (5RCTs)	Other*	CER/Regular diet	MD=-1.36 (-3.23, 0.51), P=0.15	Very low quality
Roman, 2019		666 (9RCTs)	5:2 diet/Other*	CER/Regular diet	MD=-0.31 (-1.61, 0.98), P=0.64	Moderate quality
Allaf, 2021		719 (10RCTs)	5:2 diet/ADF/Other*	CER (≤12 weeks)	MD=-0.88 (-1.76, 0.00), P=0.05	Low quality
		279 (4RCTs)	5:2 diet/ADF/Other*	CER (>12 weeks)	MD=-0.56 (-1.68, 0.56), P=0.33	Low quality
		224 (7RCTs)	5:2 diet/ADF/TRF/Other*	Regular diet (≤12 weeks)	MD=-2.88 (-3.96, -1.80), P < 0.00001	Very low quality
Guerrero, 2021		1275 (17RCTs)	5:2 diet/ADF/Other*	CER/Regular diet	Cohen's d and 95%CI= -2.42 (-3.89, -4.41) #	Moderate quality
He, 2021		850 (11RCTs)	5:2 diet/ADF/Other*	CER/Regular diet	MD=-0.95 (-1.63, -0.27), P=0.006	Moderate quality
Schwingshackl, 2021		991 (13RCTs)	5:2 diet/ADF/Other*	CER	MD= -0.55 (-1.01, -0.09), P=0.02	Moderate quality
		318 (6RCTs)	5:2 diet/ADF/Other*	Regular diet	MD=-4.83 (-5.46, -4.21), P < 0.00001	Low quality

Summary of findings from the meta-analysis for the outcome of BMI (kg/m^2)

Systematic reviews (First author, year)	Outcome	Number of participants (studies)	Type of intervention	Type of comparison	Anticipated absolute effects (95%CI), P	Quality of evidence (GRADE)
Allaf, 2021	BMI	651 (9RCTs)	5:2 diet/ADF/Other*	CER (≤12 weeks)	MD=-0.43 (-0.76, -0.10), P=0.01	Moderate quality
		279 (4RCTs)	5:2 diet/ADF/Other*	CER (>12 weeks)	MD=-0.15 (-0.58, 0.29), P=0.51	Low quality
		115 (4RCTs)	5:2 diet/ADF/TRF/Other*	Regular diet (≤12 weeks)	MD=-0.92 (-1.36, -0.48), P < 0.00001	Very low quality
Guerrero, 2021		431 (6RCTs)	5:2 diet/ADF/Other*	CER/Regular diet	Cohen's d and 95%CI=-0.38 (-2.55, -2.8) #	Moderate quality

Summary of findings from the meta-analysis for the outcome of waist circumference (cm)

Systematic reviews (First author, year)	Outcome	Number of participants (studies)	Type of intervention	Type of comparison	Anticipated absolute effects (95%CI), P	Quality of evidence (GRADE)
Cioffi, 2018	Waist circumference	385 (5RCTs)	5:2 diet/ADF/Other*	CER/ Regular diet/No control	MD= -0.17 (-1.74, 1.39), P=0.83	Very low quality
Harris and Hamilton, 2018		N/A (2RCTs)	5:2 diet/ADF/Other*	CER	WMD=-2.14 (-3.53, -0.75), P=0.002	Low quality
Roman, 2019		348 (4RCTs)	5:2 diet/Other*	CER/Regular diet	MD=-0.54 (-3.39, 2.31), P=0.71	Low quality
Allaf, 2021		557 (8RCTs)	5:2 diet/ADF/Other*	CER (≤12 weeks)	MD=-0.83 (-2.11, 0.44), P=0.20	Moderate quality
		258 (3RCTs)	5:2 diet/ADF/Other*	CER (>12 weeks)	MD=-0.66 (-2.55, 1.23), P=0.49	Very low quality
		87 (2RCTs)	5:2 diet/ADF/TRF/Other*	Regular diet (≤12 weeks)	MD=-4.19 (-6.38, -2.01), P = 0.0002	Very low quality
Guerrero, 2021		131 (8RCTs)	5:2 diet/ADF/Other*	CER/Regular diet	Cohen's d and 95%CI= -0.37 (-4.27, -4.7) #	Low quality
He, 2021		319(4RCTs)	5:2 diet/ADF/Other*	CER/Regular diet	MD=-0.53 (-2.56, 1.49), P=0.61	Low quality
Schwingshackl, 2021		641 (8RCTs)	5:2 diet/ADF/Other*	CER	MD= -0.57 (-1.56, 0.41), P=0.25	Moderate quality
		142 (2RCTs)	5:2 diet/ADF/Other*	Regular diet	MD=-1.73 (-3.69, 0.24), P=0.08	Very low quality

Summary of findings from the meta-analysis for the outcome of body composition (kg)

Systematic reviews (First author, year)	Outcome	Number of participants (studies)	Type of intervention	Type of comparison	Anticipated absolute effects (95%CI), P	Quality of evidence (GRADE)
Cioffi, 2018	FM	476 (7RCTs)	5:2 diet/ADF/Other*	CER/ Regular diet/No control	WMD=-0.23 (-1.23, 0.77), P=0.66	Moderate quality
Harris and Hamilton, 2018		N/A (2RCTs)	5:2 diet/ADF/Other*	CER	WMD=-1.38 (-2.47, -0.28), P=0.014	Very low quality
		N/A (2RCTs)	5:2 diet/ADF/Other*	Regular diet	WMD=-3.24 (-4.55, -1.92), P<0.001	Very low quality
Roman, 2019		360 (5RCTs)	5:2 diet/Other*	CER/Regular diet	MD=-0.26 (-1.14, 0.62), P=0.56	Low quality
Guerrero, 2021		840 (13RCTs)	5:2 diet/ADF/Other*	CER/Regular diet	Cohen's d and 95%CI=-0.38 (-3.25, -3.87) #	Very low quality
He, 2021		607(8RCTs)	5:2 diet/ADF/Other*	CER/Regular diet	MD=-0.65 (-1.36, 0.07), P=0.08	Moderate quality
Schwingshackl, 2021		743 (10RCTs)	5:2 diet/ADF/Other*	CER	MD=-0.66 (-1.14, -0.19), P=0.007	Moderate quality
		279 (6RCTs)	5:2 diet/ADF/Other*	Regular diet	MD=-2.54 (-3.78, -1.31), P < 0.00001	Very low quality
Cioffi, 2018	FFM	476 (7RCTs)	5:2 diet/ADF/Other*	CER/ Regular diet/No control	WMD= -0.22 (-1.01, 0.56), P=0.58	Moderate quality
Harris and Hamilton, 2018		N/A (2RCTs)	5:2 diet/ADF/Other*	CER	WMD= -0.02 (-0.80, 0.76), P=0.958	Low quality
Roman, 2019		322 (4RCTs)	5:2 diet/Other*	CER/Regular diet	MD=-0.86 (-1.62, -0.10), P=0.03	Low quality
Guerrero, 2021		797 (12RCTs)	5:2 diet/ADF/Other*	CER/Regular diet	Cohen's d and 95%CI=-0.39 (-1.51, -1.72) #	Very low quality
He, 2021		607(8RCTs)	5:2 diet/ADF/Other*	CER/Regular diet	MD= -0.39 (-0.85,0.06), P=0.09	Moderate quality

Abbreviations: ADF, alternate day fasting; BMI, body mass index; BP: blood pressure; CER, continuous energy restriction; CRP, C-reactive protein; CI, confidence interval; DBP, diastolic blood pressure; FM, fat mass; FFM, fat free mass; HDL-C: high-density lipoprotein cholesterol; HbA1c, glycated hemoglobin; IER, intermittent energy restriction; LDL-C, low-density lipoprotein cholesterol; MD, mean difference; N/A, not applicable; RCT, randomized controlled trial; SBP, systolic blood pressure; TC, total cholesterol; TG, triglycerides. WMD, weighted mean difference.

*Not IER protocols prescribed in the inclusion criteria.

#P value was not available.
