# Supplementary material for: Comparison of the Effects of Intermittent Energy Restriction and Continuous Energy Restriction among Adults with Overweight or Obesity: An Overview of Systematic Reviews and Meta-Analyses
Source: Nutrients. 2022 May 31;14(11):2315. doi: 10.3390/nu14112315 (PMC9183159; doi:10.3390/nu14112315)
Supplement: Supplementary file 1 [file nutrients-14-02315-s001.zip › Supplementary Materials S5. Quality assessment of systematic reviews by AMSTAR 2.pdf]

Supplementary Materials S5. Quality assessment of systematic reviews by AMSTAR 2

Systematic reviews (First author, year)	Q1	Q2	Q3	Q4	Q5	Q6	Q7	Q8	Q9	Q10	Q11	Q12	Q13	Q14	Q15	Q16	Overall rating
**Reviews with meta-analysis**																	
Headland, 2016	Y	PY	Y	PY	Y	N	N	Y	N	N	Y	Y	Y	Y	N	Y	Critically low
Cioffi, 2018	Y	PY	Y	PY	Y	Y	N	PY	Y	N	Y	N	N	N	Y	Y	Critically low
Harris and McGarty, 2018	Y	PY	Y	PY	Y	Y	N	Y	Y	N	N/A	N/A	N/A	N	N/A	Y	Low
Harris and Hamilton, 2018	Y	PY	Y	PY	Y	Y	Y	Y	Y	N	Y	Y	Y	Y	Y	Y	Moderate
Roman, 2019	Y	PY	Y	PY	Y	Y	N	Y	Y	N	Y	Y	Y	Y	N	Y	Critically low
Schwingshackl, 2020	Y	PY	Y	PY	Y	Y	Y	PY	Y	N	Y	Y	Y	Y	N	Y	Low
Guerrero, 2020	Y	PY	Y	PY	Y	Y	N	Y	N	N	Y	N	N	N	N	Y	Critically low
He, 2021	Y	PY	Y	PY	Y	Y	N	Y	Y	N	Y	Y	Y	Y	N	Y	Critically low
Allaf, 2021	Y	Y	Y	Y	Y	Y	Y	Y	Y	Y	Y	Y	Y	Y	Y	Y	High

**Reviews without meta-analysis**																	
Seimon, 2015	Y	PY	Y	PY	Y	Y	N	Y	N	N	N/A	N/A	N/A	N	N/A	N	Critically low
Davis, 2016	Y	PY	Y	PY	Y	Y	N	Y	Y	Y	N/A	N/A	N/A	N	N/A	Y	Low
Vitale, 2020	Y	PY	Y	PY	Y	N	N	Y	Y	N	N/A	N/A	N/A	N	N/A	Y	Low

Abbreviations: N, no; N/A, not applicable; PICOS, Population, Intervention, Comparison, Outcomes, Study design; PY, partial yes; Y, yes.

1. Did the research questions and inclusion criteria for the review include the components of PICO? (Yes/No)

2. Did the report of the review contain an explicit statement that the review methods were established prior to the conduct of the review and did the report justify any significant deviations from the protocol? (Yes/Partial Yes/No)

3. Did the review authors explain their selection of the study designs for inclusion in the review? (Yes/No)

4. Did the review authors use a comprehensive literature search strategy? (Yes/Partial Yes/No)

5. Did the review authors perform study selection in duplicate? (Yes/No)

6. Did the review authors perform data extraction in duplicate? (Yes/No)

7. Did the review authors provide a list of excluded studies and justify the exclusions? (Yes/Partial Yes/No)

8. Did the review authors describe the included studies in adequate detail? (Yes/Partial Yes/No)

9. Did the review authors use a satisfactory technique for assessing the risk of bias (RoB) in individual studies that were included in the review? (Yes/Partial Yes/No/Includes only NRSI)

10. Did the review authors report on the sources of funding for the studies included in the review? (Yes/No)

11. If meta-analysis was performed did the review authors use appropriate methods for statistical combination of results? (Yes/No/No meta-analysis conducted)

12. If meta-analysis was performed, did the review authors assess the potential impact of RoB in individual studies on the results of the meta-analysis or other evidence synthesis? (Yes/No/No meta-analysis conducted)

13. Did the review authors account for RoB in individual studies when interpreting/ discussing the results of the review? (Yes/No)

14. Did the review authors provide a satisfactory explanation for, and discussion of, any heterogeneity observed in the results of the review? (Yes/No)

15. If they performed quantitative synthesis did the review authors carry out an adequate investigation of publication bias (small study bias) and discuss its likely impact on the results of the review? (Yes/No/No meta-analysis conducted)

16. Did the review authors report any potential sources of conflict of interest, including any funding they received for conducting the review? (Yes/No)
