# Supplementary figures and images for: Comparison of the Effects of Intermittent Energy Restriction and Continuous Energy Restriction among Adults with Overweight or Obesity: An Overview of Systematic Reviews and Meta-Analyses
Source: Nutrients. 2022 May 31;14(11):2315. doi: 10.3390/nu14112315 (PMC9183159; doi:10.3390/nu14112315)

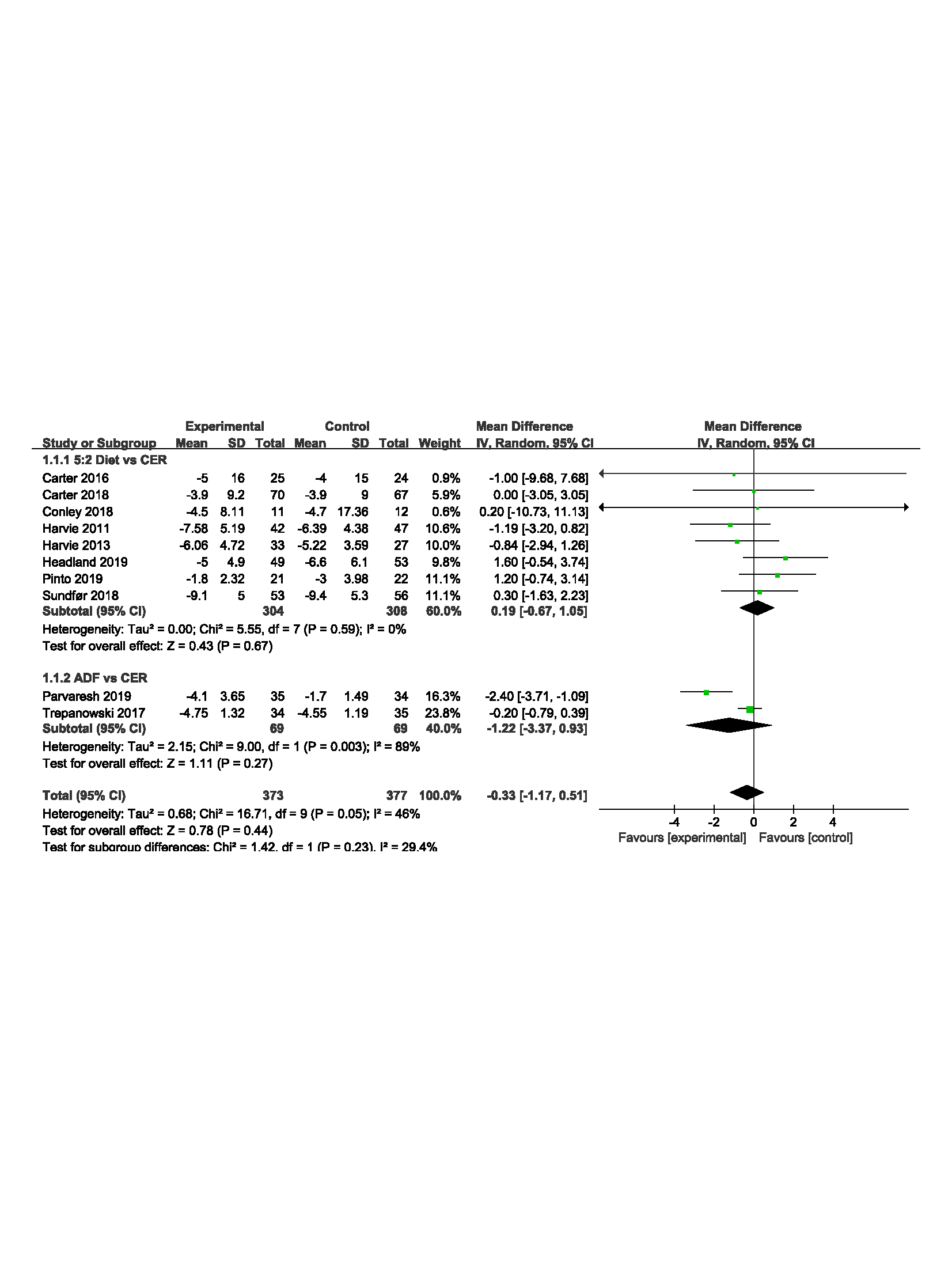

Supplement: Supplementary file 1 [file nutrients-14-02315-s001.zip › Figure S1. Forest plot for body weight in trials that compared subtypes of IER with CER.tif]

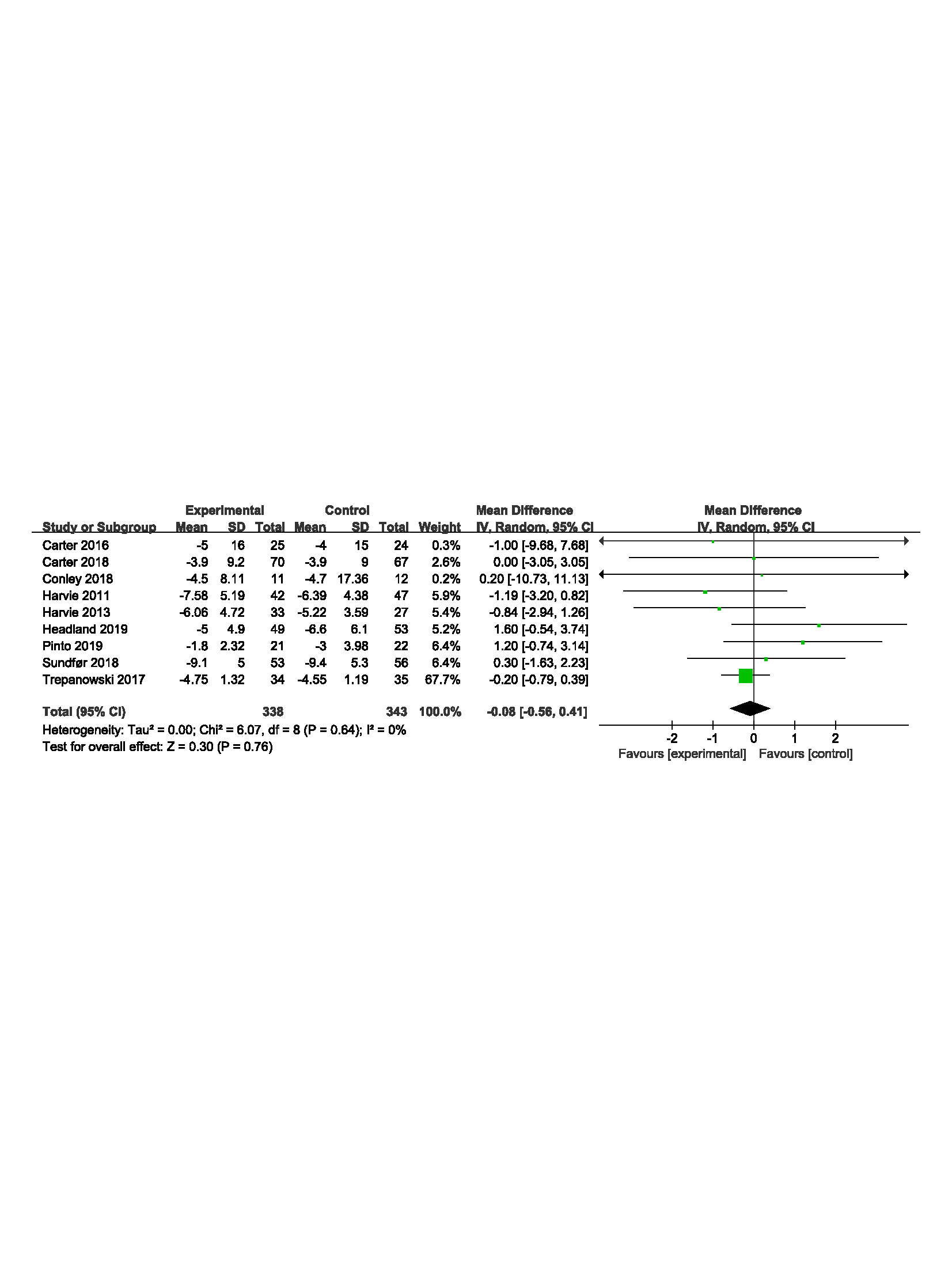

Supplement: Supplementary file 1 [file nutrients-14-02315-s001.zip › Figure S2. Forest plot of sensitivity analysis for body weight in trials that compared IER with CER.tif]

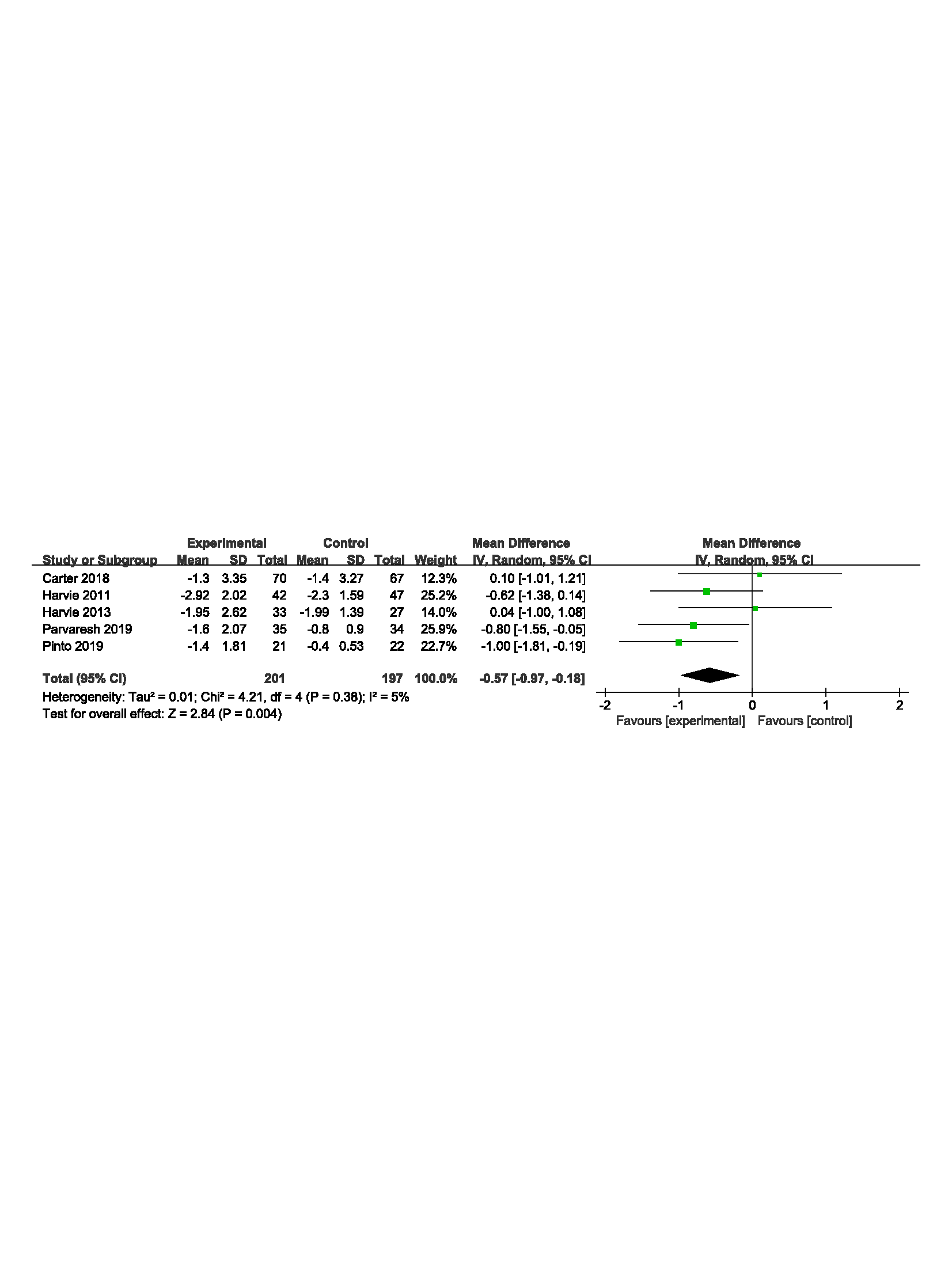

Supplement: Supplementary file 1 [file nutrients-14-02315-s001.zip › Figure S3. Forest plot of sensitivity analysis for BMI in trials that compared IER with CER.tif]

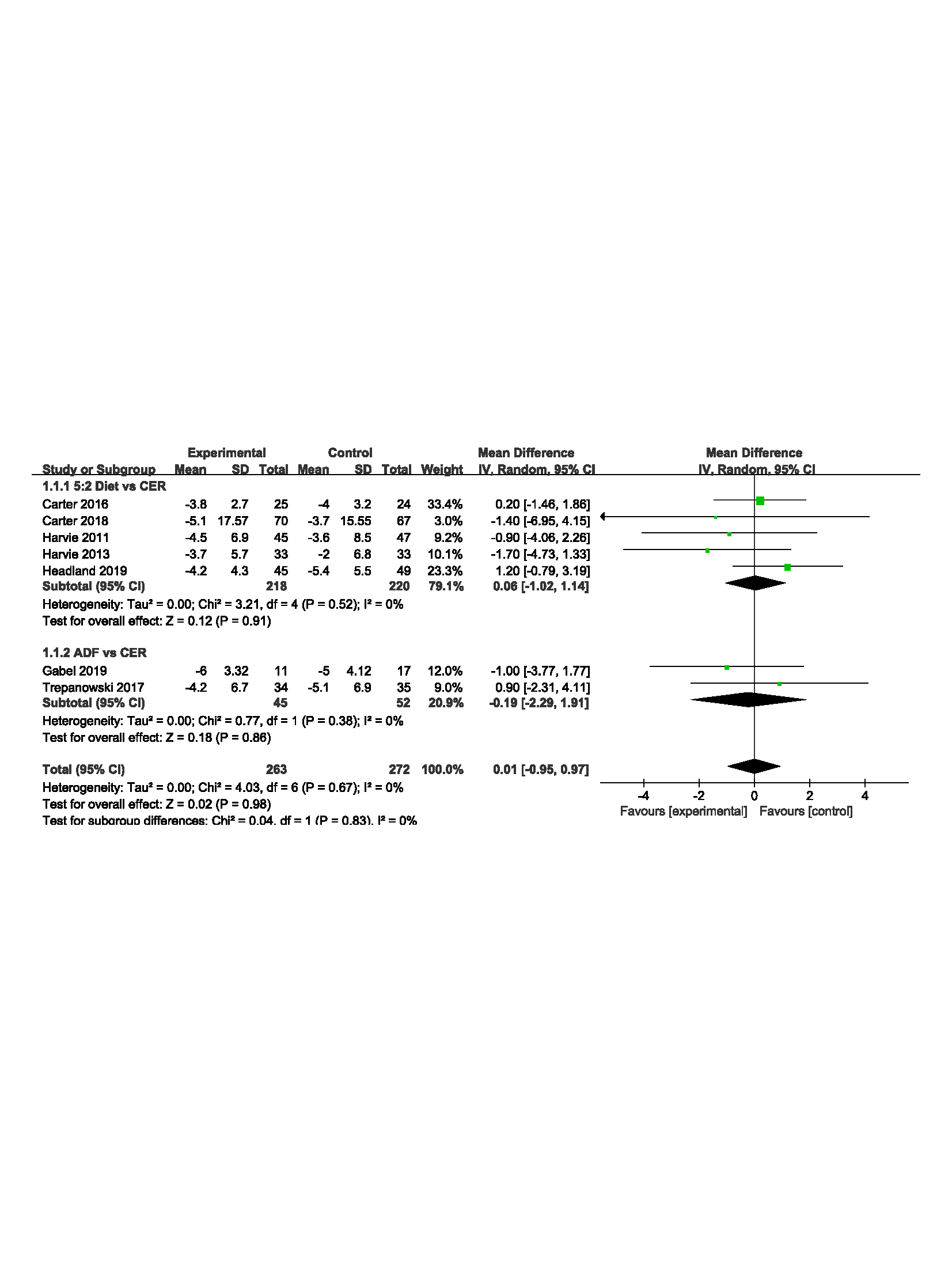

Supplement: Supplementary file 1 [file nutrients-14-02315-s001.zip › Figure S4. Forest plot for FM in trials that compared subtypes of IER with CER.tif]

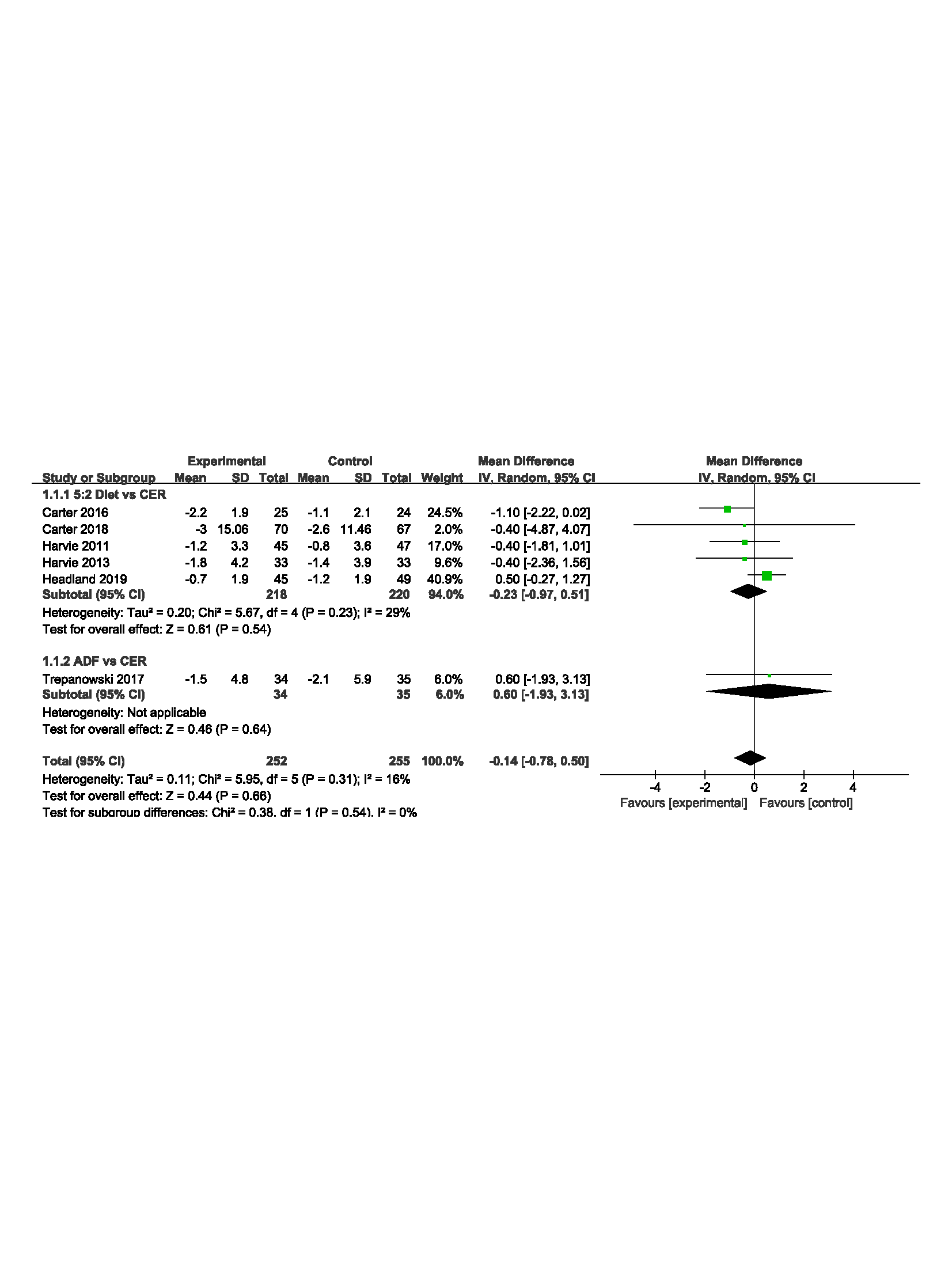

Supplement: Supplementary file 1 [file nutrients-14-02315-s001.zip › Figure S5. Forest plot for FFM in trials that compared subtypes of IER with CER.tif]
